# Prediction of Vestibular Dysfunction by Applying Machine Learning Algorithms to Postural Instability

**DOI:** 10.3389/fneur.2020.00007

**Published:** 2020-02-05

**Authors:** Teru Kamogashira, Chisato Fujimoto, Makoto Kinoshita, Yayoi Kikkawa, Tatsuya Yamasoba, Shinichi Iwasaki

**Affiliations:** Department of Otolaryngology and Head and Neck Surgery, University of Tokyo, Tokyo, Japan

**Keywords:** posturography tests, machine learning (artificial intelligence), vestibular dysfunction, Gradient Boosting Decision Tree (GBDT), hyperparameter

## Abstract

**Objective:** To evaluate various machine learning algorithms in predicting peripheral vestibular dysfunction using the dataset of the center of pressure (COP) sway during foam posturography measured from patients with dizziness.

**Study Design:** Retrospective study.

**Setting:** Tertiary referral center.

**Patients:** Seventy-five patients with vestibular dysfunction and 163 healthy controls were retrospectively recruited. The dataset included the velocity, the envelopment area, the power spectrum of the COP for three frequency ranges and the presence of peripheral vestibular dysfunction evaluated by caloric testing in 75 patients with vestibular dysfunction and 163 healthy controls.

**Main Outcome Measures:** Various forms of machine learning algorithms including the Gradient Boosting Decision Tree, Bagging Classifier, and Logistic Regression were trained. Validation and comparison were performed using the area under the curve (AUC) of the receiver operating characteristic curve (ROC) and the recall of each algorithm using K-fold cross-validation.

**Results:** The AUC (0.90 ± 0.06) and the recall (0.84 ± 0.07) of the Gradient Boosting Decision Tree were the highest among the algorithms tested, and both of them were significantly higher than those of the logistic regression (AUC: 0.85 ± 0.08, recall: 0.78 ± 0.07). The recall of the Bagging Classifier (0.82 ± 0.07) was also significantly higher than that of logistic regression.

**Conclusion:** Machine learning algorithms can be successfully used to predict vestibular dysfunction as identified using caloric testing with the dataset of the COP sway during posturography. The multiple algorithms should be evaluated in each clinical dataset since specific algorithm does not always fit to any dataset. Optimization of the hyperparameters in each algorithm are necessary to obtain the highest accuracy.

## Introduction

The postural control system in humans is maintained by muscular actions governed by the central nervous system, which integrates information from vestibular, visual, and somatosensory inputs. Posturography is a clinical technique used to measure the movement of the center of foot pressure (COP) during sway in order to analyze postural control performance in detail (1). In this posturography system, various parameters of the COP have been used to investigate vestibular disorders, central nervous system disorders and orthopedic disorders (2–4). These parameters include the velocity, path length, envelopment area, movements in the medial-lateral and/or anterior-posterior direction, amplitude of displacement, power frequency, and Romberg's ratio, which is the ratio of parameters in eyes-closed to eyes-open conditions. To statistically analyze these parameters obtained from the COP measurements, a generalized linear model has often been utilized (3).

Recently, machine learning, which is a set of computational methods that learn patterns in data without being explicitly programmed, has been utilized in the field of medicine (5). Machine learning algorithms can be roughly divided into two algorithms: a method for predicting an answer for a new case based on a dataset whose correct answer is known, and a method for classifying a dataset into multiple groups. In clinical research, the former method has frequently been adopted, and many studies have been undertaken to try to predict diseases from medical images based on an algorithm learned from a dataset corresponding multiple images with diseases (6, 7). Among various algorithms of machine learning, a convolutional neural network has been widely used as a suitable method for predicting the disease from the image, although the type of effective machine learning algorithms may differ depending on the nature of the dataset and the number of datasets to be studied.

Among the machine learning algorithms, an artificial neural network has been applied to process the parameters obtained from posturography to assess fall risk and to diagnose various balance disorders (8–10). However, the efficacy of the many other machine learning algorithms have not been evaluated in detail with regard to posturography parameters.

The current study aimed to evaluate multiple machine learning algorithms and traditional statistical algorithms to predict the presence of peripheral vestibular dysfunction from posturography parameters. We evaluated various machine learning algorithms in predicting vestibular dysfunction using a dataset of the center of pressure (COP) sway during foam posturography obtained from patients with dizziness.

## Subjects and Methods

Patients were recruited between January 2017 and November 2018 at the Balance Disorder Clinic, Department of Otolaryngology, The University of Tokyo Hospital. The study was approved by the regional ethical standards committee in the Faculty of Medicine at the University of Tokyo. The study was conducted according to the tenets of the Declaration of Helsinki, and informed consent was obtained from each participant. Patients were scheduled to undergo caloric testing before posturography. Both tests were performed on the same day. The posturography test has been taken 1 h or over after the caloric test to prevent the effect of dizziness. Caloric testing was performed in a darkened room by irrigating the external auditory canal with 2 mL ice water (4°C) for 20 s followed by aspiration of water. This method of caloric stimulation is easier to perform than bithermal irrigation with water at 30 and 44°C, and has been shown to have a high sensitivity and specificity for detecting canal paresis (CP) based on Jongkees' formula (11). Caloric nystagmus was recorded using an electronystagmograph. An abnormal caloric response was defined by either of the following criteria: (1) CP percentage >20% (12); or (2) maximum slow phase eye velocity <10°/s bilaterally (13). A total of 99 consecutive patients showed abnormal caloric responses. We excluded 14 patients who had non-vestibular diseases that could cause postural instability: brainstem hemangioma (*n* = 2), migraine (*n* = 2), psychogenic disorder (*n* = 2), meningitis (*n* = 1), multiple neuropathy (*n* = 1), neurosarcoidosis (*n* = 1), orthostatic disturbance (*n* = 1) and disturbance of deep sensation (*n* = 1). Thus, 85 patients (41 men, 44 women) were enrolled in this study. The mean age (±standard deviation), the mean height, and the mean weight of the 85 patients were 52.6 (±15.4) years, 161.7 (±7.7) cm and 59.7 (±15.3) kg, respectively.

Of the 85 patients, 50 patients were diagnosed as having unilateral peripheral vestibulopathy, with etiologies of Meniere's disease (*n* = 13), acoustic tumor (*n* = 12), vestibular neuritis (*n* = 8), benign paroxysmal positional vertigo (*n* = 6), Ramsay-Hunt syndrome (*n* = 3), cholesteatoma (*n* = 2), delayed endolymphatic hydrops (*n* = 2), sudden deafness with vestibular dysfunction (*n* = 2), otosclerosis (*n* = 1), and temporal bone fracture (*n* = 1). The other 31 patients with unilateral peripheral vestibulopathy could not be diagnosed as having an established clinical entity. Four patients showed bilateral abnormal caloric responses, with an etiology of idiopathic bilateral vestibulopathy (*n* = 2) or another etiology (*n* = 2). The CP percentage ranged from 20 to 100, and the average (±standard deviation) was 54.0 (±27.5).

We enrolled 163 healthy control subjects (84 men, 79 women) in the present study. Subjects in the control group have no symptoms of dizziness, no dizziness in the past, and no problem with walking. The mean age (±standard deviation), the mean height, and the mean weight of the 163 healthy control subjects were 48.7 (±22.5) years, 160.6 (±11.9) cm, and 59.4 (±11.7) kg, respectively.

We used a Gravicorder G-5500 (Anima Co. Ltd., Tokyo, Japan) with/without a foam rubber layer on the posture platform (Nagashima Medical Instruments, Tokyo, Japan). The posture platform contains vertical force transducers to determine instantaneous fluctuations in the COP at a sampling frequency of 20 Hz. The sway path of the COP was obtained from these data. The foam rubber material was made of natural rubber, with a tensile strength of 2.1 Kgf/cm^2^, an elongation stretch percentage of 110%, a density of 0.06 g/cm^3^, and a thickness of 3.5 cm. Two-legged stance tasks were performed with both arms at the subject's side under four conditions: eyes open or eyes closed, with or without the foam rubber. The distal ends of the big toes were positioned 45 degrees apart with the heels of both feet close to each other. The recording time was 60 s or until the subject required assistance to prevent falling. In the eyes-open condition, the subjects were asked to watch a small red circle 2 m away from where they were standing in a quiet, well-lit room. We measured the mean velocity of movement of the COP for 60 s, which was termed “the velocity,” and the envelopment area traced by the movement of the COP, which was termed “the area.” These parameters can indirectly reflect the function of the peripheral and central vestibular system due to the reduction of visual and somatosensory inputs.

We estimated the power spectrum of the acceleration signal for the anterior-posterior (AP) and the medial-lateral (ML) axes by using the maximum entropy method (MEM), which is superior to a fast Fourier transform for analyzing relatively short samples. Frequency resolution was set at 0.001 Hz, and the lag value was set at 120. The area under the curve (AUC) of the power spectral density (PSD) of the COP were calculated for each axis across three frequency ranges: between 0.02 and 0.1 Hz (low-frequency range, LF-AUC), between 0.1 and 1 Hz (middle-frequency range, MF-AUC) and between 1 and 10 Hz (high-frequency range, HF-AUC). The sum of these individual AUCs was also calculated (total AUC) (14).

The dataset included the presence of peripheral vestibular dysfunction (VD) evaluated with the method above, the subject's age and the posturography parameters, which were the velocity, the area, the MF-AUC of the AP and ML axes and the total AUC of the AP and ML axes.

**Figure 1:**
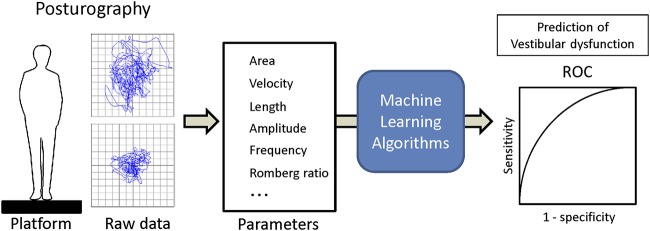
Overview of the machine learning analysis from posturography data.

Training and analysis were completed using Python 3.5 with scipy 0.18 and scikit-learn 0.18, and R version 3.4.4 (15). The applied supervised machine learning algorithms were ensemble methods (adaptive boosting classifier, bagging classifier, extra trees classifier, gradient boosting classifier, random forest classifier), support vector classification (SVC) [c-support vector classification (SVC), nu-support vector classification (Nu SVC)], decision trees (decision tree classifier, extra tree classifier), multi-layer perceptron classifier (MLPClassifier, neural network, deep learning) and generalized linear models [logistic regression, stochastic gradient descent (SGD) classifier]. The dataset was separated into the training dataset and the validation dataset with the split ratio of 80 and 20%. Then each machine learning algorithm was trained with the training dataset, and vestibular dysfunction in the validation dataset was predicted using trained algorithms from the validation dataset. The recall and the AUC of ROC were calculated (Figure 1). The validation and comparison of the algorithms were performed using K-fold cross-validation with the Wilcoxon signed-rank test, and *p* < 0.05 was considered to be significant (16, 17).

## Results

**Figure 2:**
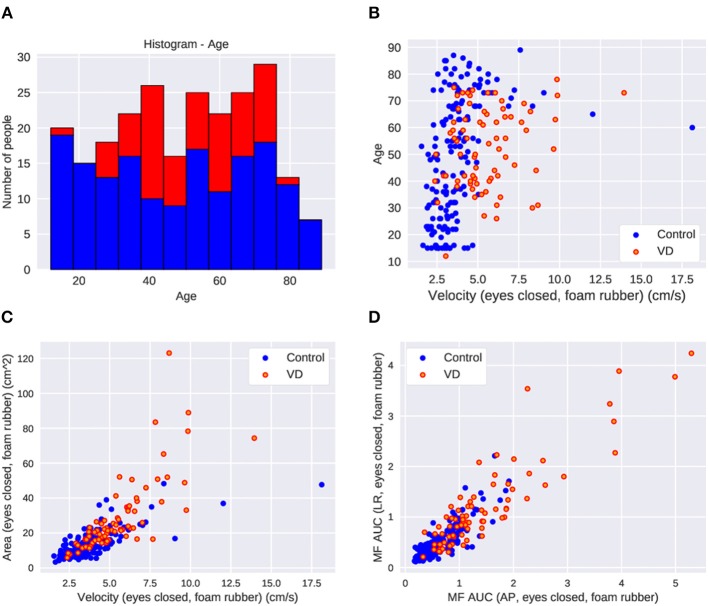
The parameter distribution of the dataset in control and patients with vestibular dysfunction (VD). **(A)** Histogram of age. **(B)** Scatter plot of age and velocity of COP in the eyes-closed foam rubber condition. Greater VD cases at higher velocities. **(C)** Scatter plot of area and velocity in the eyes-closed foam rubber condition. More VD cases where the area and velocity are large. **(D)** Scatter plot of middle frequency AUC in the back and forth direction and middle frequency AUC in the left and right direction in the eyes-closed foam rubber condition. There are more VD patients in cases where each parameter is large.

All of the healthy control subjects were able to complete posturography tests. The 10 patients were unable to stand for 60 s in eyes closed/foam rubber condition and to complete the exam. Eight of them were patients with unilateral peripheral vestibulopathy, and the other two were patients with bilateral peripheral vestibulopathy. We analyzed the data obtained from the remaining 75 patients. The basic physical parameters and posturography results of 75 patients with peripheral vestibular dysfunction and 163 healthy controls are shown in Table 1 and Figure 2. There were no significant differences in age, height, or body weight between the patients and the healthy controls (*p* > 0.05, respectively). On the other hand, the velocity and the area in the eyes-closed condition on the foam rubber were significantly greater in patients compared to controls (*p* < 0.0001).

**Table 1:** The basic physical parameters and posturography results.

**Parameters**	**Average** **±** **standard deviation**		**Significant difference**
	**Healthy subjects**	**Patients with CP**	
Age	48.7 ± 22.5	52.6 ± 15.4	*p* > 0.05
Height	160.6 ± 11.9 cm	161.7 ± 7.7 cm	*p* > 0.05
Body weight	59.4 ± 11.7 k	59.7 ± 15.3 k	*p* > 0.05
MF-AUC of AP axis with eyes closed and with rubber	0.6 ± 0.3 cm^2^	1.5 ± 1.0 cm^2^	*p* < 0.0001
MF-AUC of LR axis with eyes closed and with rubber	0.5 ± 0.3 cm^2^	1.2 ± 0.9 cm^2^	*p* < 0.0001
Total AUC of AP axis with eyes closed and with rubber	1.0 ± 0.6 cm^2^	1.9 ± 1.2 cm^2^	*p* < 0.0001
Total AUC of LR with eyes closed and with rubber	0.7 ± 0.5 cm^2^	1.6 ± 1.1 cm^2^	*p* < 0.0001
Velocity with eyes closed and with rubber	3.7 ± 1.8 cm/s	5.6 ± 2.1 cm/s	*p* < 0.0001
Area with eyes closed and with rubber	13.6 ± 7.8 cm^2^	28.9 ± 20.7 cm^2^	*p* < 0.0001

**Figure 3:**
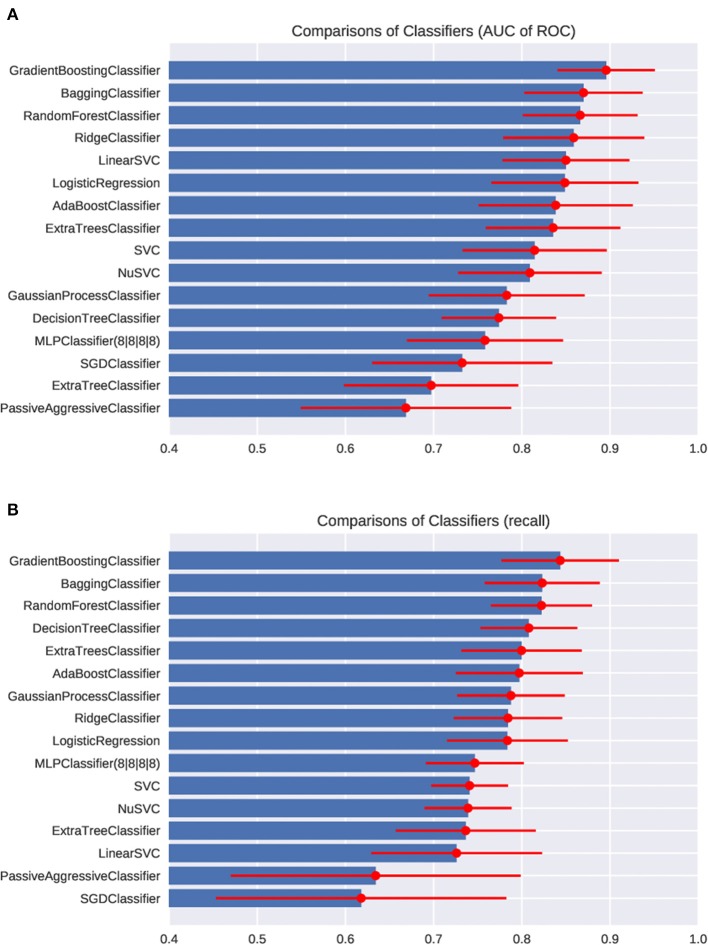
Comparisons of classifiers in the AUC of ROC **(A)** and the recall **(B)**.

**Figure 4:**
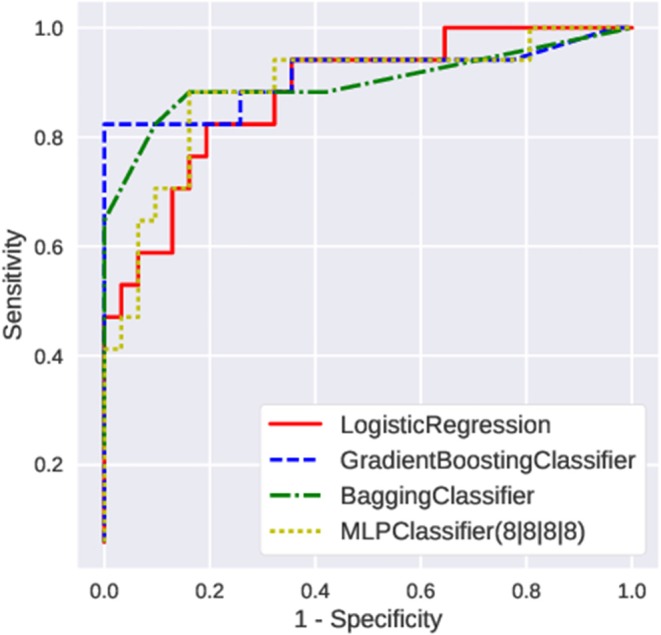
The ROC curve of typical machine learning algorithms.

First, we fed the age and the posturography data into the various machine-learning algorithms, and compared the AUC and the recall regarding the presence of peripheral vestibular dysfunction (Figure 3). Among the algorithms tested, the gradient boosting classifier showed the highest AUC (0.89 ± 0.05) as well as the highest recall (0.82 ± 0.06). Both the AUC and the recall were significantly higher in the gradient boosting classifier than those of the logistic regression (AUC: 0.85 ± 0.06; recall: 0.78 ± 0.06). The AUC and the recall of the SVC (AUC: 0.81; recall: 0.73) and MLPClassifier (AUC: 0.76; recall: 0.73) were significantly lower than those of the logistic regression (*p* < 0.05). The recall of the bagging classifier (0.81 ± 0.05) and random forest classifier (0.81 ± 0.05) were also significantly higher compared to the logistic regression (*p* < 0.05). The comparisons of the AUC and the recall of all algorithms tested are shown in Supplementary Table 1. The ROC curves of the Gradient boosting classifier, Bagging classifier, logistic regression, and MLPClassifier are shown in Figure 4. The ROC curves of the all algorithms tested are shown in Supplementary Figure 2, and the ROC curve of logistic regression using typical posturography parameter are shown in Supplementary Figure 3. The sensitivity as well as the specificity of the gradient boosting classifier, the bagging classifier and the MLPClassifier were higher than those of the logistic regression.

**Figure 5:**
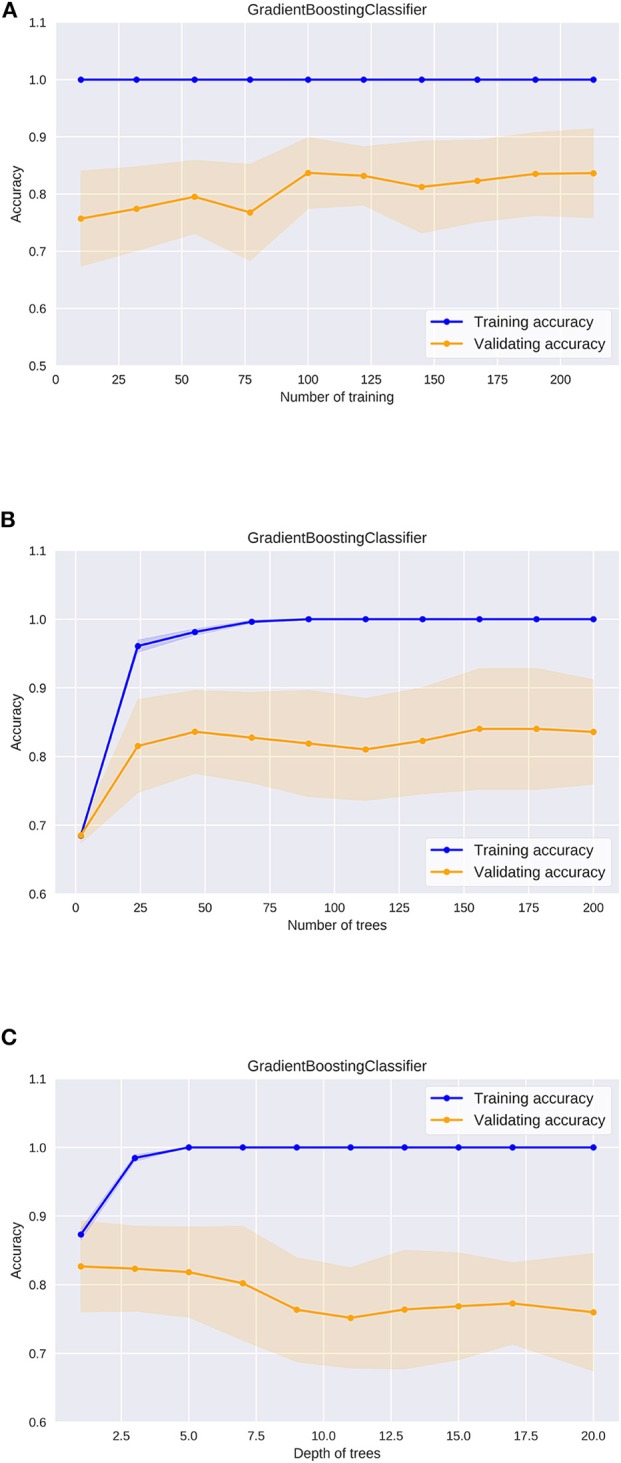
The learning curve of the gradient boosting classifier **(A)**, the validation curve of the number of trees in the gradient boosting classifier **(B)**, the validation curve of the depth of trees in the gradient boosting classifier **(C)**.

Then, we evaluated the learning curve of the Gradient Boosting Classifier, which showed the best performance (Figure 5A). The training accuracy of the gradient boosting classifier was almost 1, and the validating accuracy tended to improve up to 0.85 over 100 training instances. This result indicates that higher predictability could be expected in the gradient boosting classifier if the dataset is much larger.

In utilizing machine learning algorithms, it is important to pay attention to the risk of overfitting. Changes in accuracy during the optimization process must be carefully evaluated for each hyperparameter. In ensemble learning algorithms including the gradient boosting classifier, some models are built from multiple decision trees (Supplementary Figure 1). We evaluated how the accuracy changes as the size and the depth of the decision trees were adjusted in the gradient boosting classifier. Regarding the number of decision trees, the validating accuracy was the highest when the number was about 50, while it tended to decrease when the number was more than 100 (Figure 5B). The training accuracy was 1 when the number was more than 100, and the gradient boosting classifier algorithm was the most efficient model to predict peripheral vestibular dysfunction in this dataset. Regarding the depth of the decision tree, the validating accuracy was the highest at around 3, and the accuracy tended to decline as the tree became deeper (Figure 5C). The training accuracy was 1 when the depth was more than 5, and the gradient boosting classifier algorithm was the most efficient model to predict peripheral vestibular dysfunction in this dataset. In the optimum parameter search of this dataset, the accuracy was the highest when depth was 2 and the number of decision trees was 45. These results suggest that selecting optimal hyperparameters is an important process in utilizing machine learning algorithms.

## Discussion

In the present study, we have evaluated multiple machine learning algorithms to predict vestibular dysfunction from datasets obtained from posturography. We have shown that the ensemble learning algorithms such as the gradient boosting classifier and bagging classifier can predict vestibular dysfunction better than the generalized linear models. The prediction ability of these ensemble learning algorithms are expected to be higher than that of traditional statistical models.

Machine learning has recently become utilized in the field of medicine (5, 18), including for the detection of hepatocellular carcinoma (19), the prediction of urinary tract infections in the emergency department (20), the prediction of hip fractures (21), the diagnosis of diabetic retinopathy (22), and the prediction of heart failure (23). Machine learning is also applied in the field of neurotology where it has been evaluated for use as a new diagnostic posturographic tool for disorders of stance, with an overall sensitivity and specificity of about 0.9 (8). The vestibulo-ocular reflex rotational test has been evaluated using machine learning for the assessment of vestibular function and the accuracy was 93.4% (24). Machine learning is a promising diagnostic tool for neurological disorders.

The gradient boosting classifier was the best algorithm in our datasets. This algorithm has recently been studied in varied medical fields (20, 21, 25, 26), and has shown highly predictive performance. Other algorithms including SVC (24, 27–29), decision trees (30), genetics-based algorithms (31), and the multi-layer perceptron classifier (32) did not show the best predictive performance in our datasets. The reason for the high predictive score of the gradient boosting classifier is that this classifier identifies the shortcomings of weak learners (multiple decision trees) in the loss function (which measures the fitting of the data to the model) to optimize the model. The neural network algorithm which has been widely adopted in the research of image-based diagnosis (6, 7) was not an effective method. The reason for this was that the number of cases in our dataset was not big enough to sufficiently train the model (33). The multiple algorithms should be evaluated individually with each clinical dataset because a specific algorithm does not necessarily fit any dataset (34, 35).

The hyperparameters of the machine-learning algorithms are set values which can greatly affect the performance of the prediction model (34–36). Thus, optimization of the hyperparameters is important for achieving the best prediction results from machine learning algorithms. Because there is no mathematical expression to calculate the optimum hyperparameters, the calculation of the optimum hyperparameters is simply based on the empirical method or the exploratory method, in which various values are applied to numerous model hyperparameters to obtain the maximum prediction rate. The AUC of ROC and the recall vary depending on the hyperparameters of the algorithm, and thus it is important to search for the optimum hyperparameters. Each machine learning algorithm has multiple hyperparameters that can be adjusted to obtain better prediction performance.

Some clinical parameters which have not been determined to be effective for disease prediction by conventional statistical methods may be effective for disease prediction by bundling them with other data and processing with machine learning algorithms.

There are some limitations in our study. First, the main explanatory variables that were used to predict vestibular dysfunction may change depending on the feature of the datasets, and we need to prepare every parameter in each evaluation. Second, the study was limited to the size of the datasets. The optimum algorithm will differ depending on the numerous aspects of the datasets. Third, not all hyperparameters were evaluated in this study because calculation of all hyperparameters is extremely resource consuming. Other advanced methods of searching for the optimum hyperparameters are needed to evaluate more types of algorithms.

In this study, the vestibular function was evaluated by caloric test only because other reliable vestibular function tests including video Head Impulse Test (vHIT) was not available. The database including further vestibular functional studies will provide more clinically useful tools for vestibular diagnosis.

## Conclusion

The ensemble learning algorithms including the gradient boosting classifier and bagging classifier can predict vestibular dysfunction as identified using caloric testing from the datasets better than traditional regression algorithms.

Because the human being is a complex biological system, machine learning algorithms can build better classifiers than traditional linear classifiers to predict diagnostic values from clinical data and can be a useful tool to investigate the information in the datasets.

## Data Availability Statement

The datasets generated for this study are available on request to the corresponding author.

## Ethics Statement

The studies involving human participants were reviewed and approved by the regional ethical standards committee in the Faculty of Medicine at the University of Tokyo. Written informed consent to participate in this study was provided by the participants' legal guardian/next of kin.

## Author Contributions

TK, CF, and SI conceived of the study, conducted the experiments, wrote the manuscript, and edited the manuscript for content. MK conducted the experiments and edited the manuscript for content. YK provided statistical advice, performed statistical analysis, and edited the manuscript for content. TY conceived of the study, supervised interpretation of data, and edited the manuscript for content.

### Conflict of Interest

The authors declare that the research was conducted in the absence of any commercial or financial relationships that could be construed as a potential conflict of interest.

## Supplementary Material

Supplementary Figure 1 Examples of decision trees constituting the ensemble learning algorithm.

Supplementary Figure 2 The ROC curve of each machine learning algorithms.

Supplementary Figure 3 The ROC curve of logistic regression using single posturography parameter. The velocity (eyes closed, foam rubber) and the area (eyes closed, foam rubber) were analyzed.

Supplementary Table 1 The comparison of each machine learning algorithms (^*^*p* < 0.05).
