# Supplementary material for: Prediction of Vestibular Dysfunction by Applying Machine Learning Algorithms to Postural Instability
Source: Front Neurol. 2020 Feb 5;11:7. doi: 10.3389/fneur.2020.00007 (PMC7013037; doi:10.3389/fneur.2020.00007)
Supplement: Supplementary Figure 1 — Examples of decision trees constituting the ensemble learning algorithm. [file Data_Sheet_1.pdf]

Supplementary Figures

Supplementary Figure 1

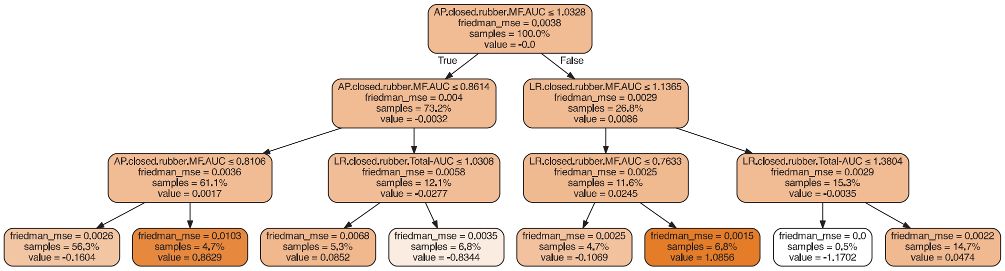

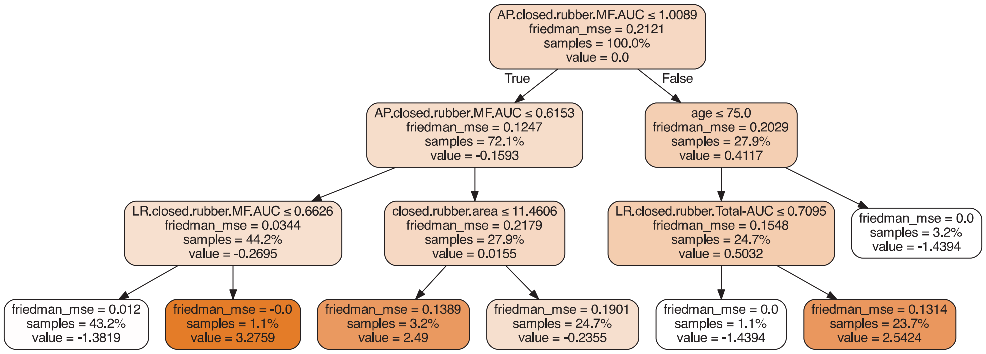

Supplementary Figure 2

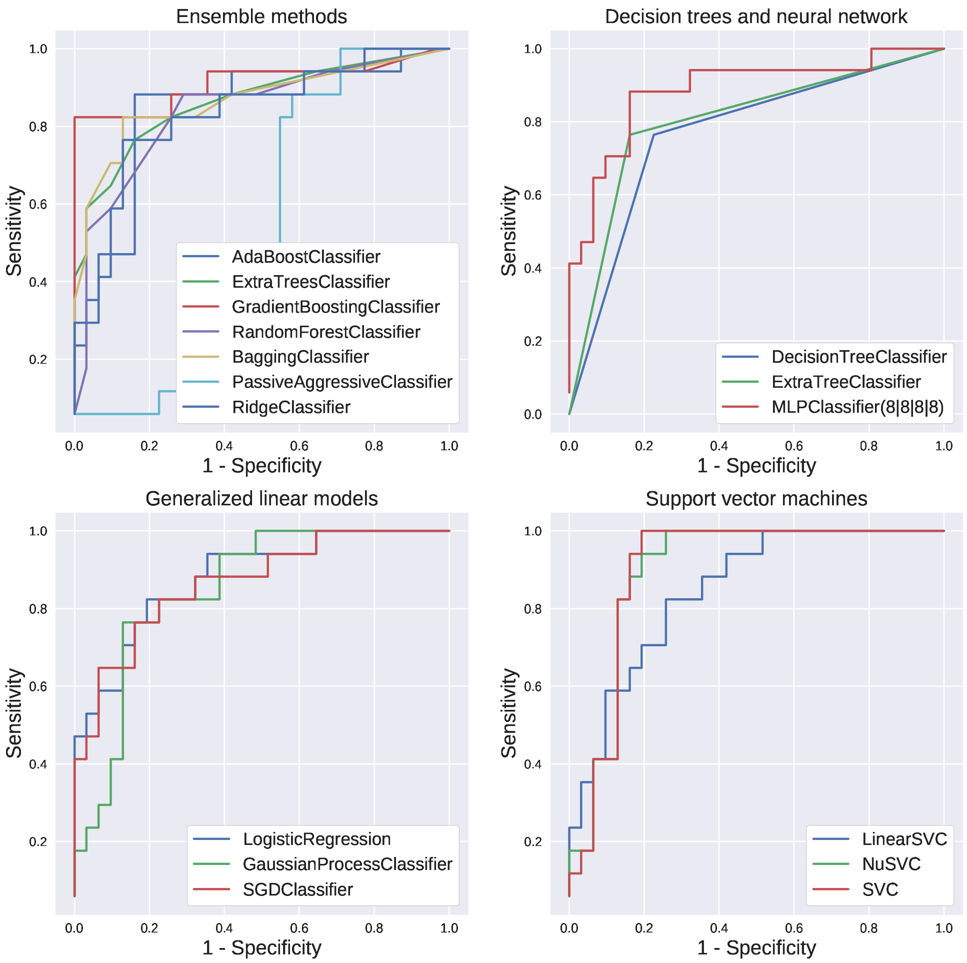

Supplementary Figure 3

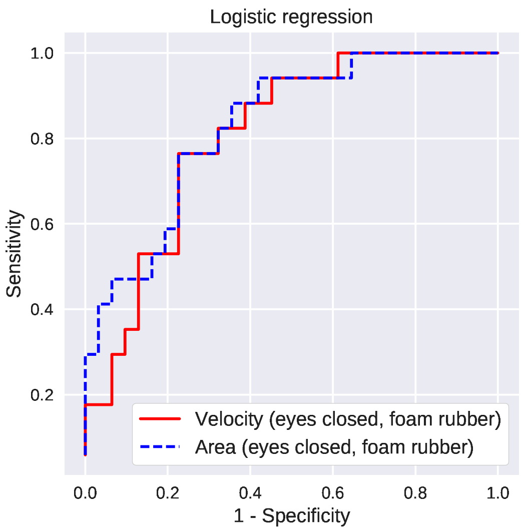

Supplementary Table 1

A

0	GradientBoostingClassifier
1	BaggingClassifier
2	RandomForestClassifier
3	RidgeClassifier
4	LinearSVC
5	LogisticRegression
6	AdaBoostClassifier
7	ExtraTreesClassifier
8	SVC
9	NuSVC
10	GaussianProcessClassifier
11	DecisionTreeClassifier
12	MLPClassifier(8|8|8|8)
13	SGDClassifier
14	ExtraTreeClassifier
15	PassiveAggressiveClassifier

AUC	0	1	2	3	4	5	6	7	8	9	10	11	12	13	14	15
0	-	*	*	*	*	*	*	*	*	*	*	*	*	*	*	*
1	-	-					*	*	*	*	*	*	*	*	*	*
2	-	-	-				*	*	*	*	*	*	*	*	*	*
3	-	-	-	-				*	*	*	*	*	*	*	*	*
4	-	-	-	-	-				*	*	*	*	*	*	*	*
5	-	-	-	-	-	-				*	*	*	*	*	*	*
6	-	-	-	-	-	-	-			*	*	*	*	*	*	*
7	-	-	-	-	-	-	-	-			*	*	*	*	*	*
8	-	-	-	-	-	-	-	-	-	*	*	*	*	*	*	*
9	-	-	-	-	-	-	-	-	-	-	*		*	*	*	*
10	-	-	-	-	-	-	-	-	-	-	-			*	*	*
11	-	-	-	-	-	-	-	-	-	-	-	-		*	*	*
12	-	-	-	-	-	-	-	-	-	-	-	-	-		*	*
13	-	-	-	-	-	-	-	-	-	-	-	-	-	-		*
14	-	-	-	-	-	-	-	-	-	-	-	-	-	-	-	
15	-	-	-	-	-	-	-	-	-	-	-	-	-	-	-	-

B

0	GradientBoostingClassifier
1	BaggingClassifier
2	RandomForestClassifier
3	DecisionTreeClassifier
4	ExtraTreesClassifier
5	AdaBoostClassifier
6	GaussianProcessClassifier
7	RidgeClassifier
8	LogisticRegression
9	MLPClassifier(8|8|8|8)
10	SVC
11	NuSVC
12	ExtraTreeClassifier
13	LinearSVC
14	PassiveAggressiveClassifier
15	SGDClassifier

recall	0	1	2	3	4	5	6	7	8	9	10	11	12	13	14	15
0	-	*	*	*	*	*	*	*	*	*	*	*	*	*	*	*
1	-	-				*	*	*	*	*	*	*	*	*	*	*
2	-	-	-			*	*	*	*	*	*	*	*	*	*	*
3	-	-	-	-						*	*	*	*	*	*	*
4	-	-	-	-	-					*	*	*	*	*	*	*
5	-	-	-	-	-	-				*	*	*	*	*	*	*
6	-	-	-	-	-	-	-			*	*	*	*	*	*	*
7	-	-	-	-	-	-	-	-		*	*	*	*	*	*	*
8	-	-	-	-	-	-	-	-	-	*	*	*	*	*	*	*
9	-	-	-	-	-	-	-	-	-	-					*	*
10	-	-	-	-	-	-	-	-	-	-	-				*	*
11	-	-	-	-	-	-	-	-	-	-	-	-			*	*
12	-	-	-	-	-	-	-	-	-	-	-	-	-		*	*
13	-	-	-	-	-	-	-	-	-	-	-	-	-	-	*	*
14	-	-	-	-	-	-	-	-	-	-	-	-	-	-	-	
15	-	-	-	-	-	-	-	-	-	-	-	-	-	-	-	-
